# Supplementary material for: Cis and trans effects differentially contribute to the evolution of promoters and enhancers
Source: Genome Biol. 2020 Aug 20;21:210. doi: 10.1186/s13059-020-02110-3 (PMC7439725; doi:10.1186/s13059-020-02110-3)
Supplement: Supplementary file 5 — Additional file 5. Review history. [file 13059_2020_2110_MOESM5_ESM.docx]

**Review history**

**First round of review**

**Reviewer 1**

This paper uses MPRA to compare activity of fragments overlapping transcription start sites from human and mouse in both human and mouse embryonic stem cells. From these data, divergence in both cis- and trans-regulation was inferred. I think this paper is a strong contribution to the field. It was also the most refreshing paper I've read in quite some time; it is beautifully and clearly written throughout (with the few exceptions noted below), making what can be a complicated topic accessible to a broad audience. I want to thank the authors for this care in preparing their work. A concrete example of this clarity is explaining the numbers of how many fragments they started and ended up with and how they got from one to the other. This degree of clarity is rare and greatly appreciated.  Figures are also nicely constructed and convey information clearly.

Despite my strong enthusiasm for this work, I take issue with two core conclusions presented, as described in more detail below. I also offer suggestions for other ways to improve the work.

Primary critiques:
1. I do not think that the conclusion stated prominently in the title, abstract, figure 6 title, and discussion, that these data argue that compensatory cis and trans effects are "rare" at individual regulatory elements and in contrast to other data in the field, is well-supported by the data. First, overall, 40% of elements affected by both cis and trans divergence showed compensatory effects. While 40% is not the majority, I would also not describe it as "rare". Second, and perhaps even more important given the way this result is presented as being in conflict with previously published work, when only the data for conserved mRNAs are considered (which is the most comparable to many of the previous RNA-seq based studies that drew conclusions about compensatory evolution), the percentage of genes with compensatory effects increases to over 50% (~65%? eyeballing last bar in Figure 6C). I would certainly not call that rare. And it looks consistent to broad conclusions from prior work to me. Finally, these conclusions are based on 10s of regulatory elements in this study, but thousands of genes in the prior work. Indeed, the sentence on page 16, line29/30 says "Conversely, conserved lncRNA and mRNA TSSs showed mostly compensatory effects". I find this in stark contrast to the title "… reveals a lack of cis-trans compensation". Especially since I think most people thinking about gene expression are thinking about expression of mRNAs (and maybe lncRNAs) much more than eRNAs. Pg 18, lines 29-32 is one example of a conclusion I don't think is consistent with the mRNA data presented, which is the subset of the current data most similar to prior work studying effects at the whole-gene level.

2. I also take issue with the strength of the data (at least as I understand it) underlying the claims about redundant regulatory elements.  Figure 6G-H take nearby (within 1Mb) additional regulatory elements (enhancers and TSSs) into account, finding that there is less compensatory divergence within one of the elements they test when there are other elements nearby. The then propose that this is because the neighboring elements might help regulate the gene and provide another mechanism for compensation. I have no problem with presenting this model as speculation (as is done at some points), but on pg 16, line59 an assumption is made that the nearby FANTOM5 regulatory elements being considered are redundant with the focal fragment that I do not see justified. That is, there might be neighboring elements, but most might NOT be redundant, which undermines the model presented.  Pg. 18, line 45 is an example of this: "we find that highly redundant regulatory elements are less likely to show compensatory…" To me, "redundant regulatory elements" implies something like shadow enhancers, that have very similar activity in space, time, and expression level. But the data used for this analysis does not seem to take the activity of nearby regulatory elements into account, so I don't think saying "redundant" is justified. This is also a key feature in the model presented in Figure 7. To be clear, I think it is fine to present this model, but the assumptions, and the appropriateness of the data used to test them, should be stated more explicitly.


Other comments:

1. The definitions used for cis and trans in the abstract and introduction are not correct. For example, take this sentence from the abstract:  "Whereas cis effects are caused by genetic variants in close proximity to the target gene, trans effects are due to distal genetic variants that affect diffusible elements such as transcription factors". Although the proximaly/distal characterization is often used to classify eQTL as likely to be cis or trans acting, it does not actually define the effects of these variants.  cis-effects are those that arise from a genetic variant affecting expression of a gene located on the same chromosome (cis = same side), and trans-effects arise from a genetic variant that can affect expression of a gene on a different (including the homologous) chromosome. The distinction between cis and trans also doesn't have to do with linkage disequilibrium, as mentioned in the introduction, although it is true that cis variants are more likely to be in LD because they are more likely to be close to the affected gene, but a variant being in LD with the affected gene is not sufficient to show that it is cis acting. It is possible for a variant to be in LD with the affected gene and act in trans as well as for a variant not to be in LD with the affected gene but to still act in cis. Given that this study doesn't use proximal/distal characterization or rely on LD to classify effects as cis or trans, I suggest you simply eliminate these terms from the manuscript to avoid confusion. (And yes, I realize that other published papers also unfortunately use similarly incorrect and/or imprecise definitions….)

2. Citations: Key papers foundational for this work are not cited. For example, Wilson et al. (2008) Science, in which mice carrying human chromosomes were used to disentangle cis- and trans-regulatory effects is very related to this work, yet not cited. In addition, a review paper (Signor et al) is cited in the introduction for the method rather than the original Wittkopp et al. (2004) paper where it was first described. In general, I think it is fine to cite a review when a point is made that is drawn from a large collection of studies, but for statements where the evidence comes from a small number (1-5?) of original research papers, I think it is more appropriate to cite the original works. Also, in the opening sentence, although the King and Wilson 1975 paper is often credited with proposing the importance of transcriptional regulation for evolutionary divergence, Britten and Davidson lay this argument out first and more clearly in their 1969 paper.  

3. Figure 2C - I would love to see a figure added to the main text that directly compares the magnitude of the cis effect measured in hESC to that measured in mESC for the same fragment. For example, a scatterplot, with one axis expression in hESC, the other expression in mESC, and each point the cis-effect measured for each fragment in the two cell types.

4. Page 6, Line 8 "mimicked endogenous activity levels" is a bit misleading. I initially thought you had compared expression driven by each fragment to that driven by the native gene. But, then realized you were just talking about the endogenous levels at the level of biotype group (eRNA, lncRNA, mRNA) rather than individual genes. Please rephrase.

5. Pg 10, line30 TYPO: "the human BCAR3-AS1 TSS and region and the region shown in Figure 3)"

6. Pg 12,line 6 TYPO: "may allow for them to absort trans effects at minimal fitness costs."

7. Pg. 18, line 18 - please explain more what you mean by "…larger accumulation of trans effects in eRNAs is likely due to their higher redundancy".

8. Pg 19, last paragraph - one additional caveat to this study should be mentioned: that the activity of regulatory elements was assayed from transfected plasmids rather than elements integrated into the genome functioning in their native genomic context. This difference likely affects the activity of the elements compared to the context in which they evolved.

9. Reference list: Danko et al is shown as reference 4 and 13.

10. Pg 3 line 22 - text says you need F1 hybrids for this analysis, but you also need parents to infer trans-regulatory differences with this strategy.

11. Pg 3, line 27-31 - oversimplifies and misrepresents the literature on cis- and trans-regulation and compensation; please go back to original papers for this work to look for intricacies that should be more faithfully represented and cited here (original research, rather than review).

12. Pg 6, line 24 - please include in the main text how the 1644 active sequences are distributed among eRNAs, lncRNAs, and mRNAs

13. Figure 2 B make X-axis labels consistent in the mESC and hESC plots. Also adjust transparency of points to make grey points more visible.

14. Pg 12, line 4 - There should be citations to support the claim that "enhancers are often redundant". I don't think the literature as a whole supports the "often" part of this sentence. I'd say "can be" or "are sometimes" redundant is a more accurate statement of our understanding. See literature on shadow enhancers and redundant enhancers.

15. Pg 14, line 7 & line21 - The language to describe the set sizes of TFs is confusing here. "We found 137 TFs were significantly associated with trans effects" & "Of the 120 TFs  we found to be associated with trans effects". I assume the second number reflects the final set size after including the RNAseq data. The section seems to just need a lead in clause to clarify why this number has changed.

**Reviewer 2**

In this study, the authors systematically performed Massively Parallel Reporter Assays (MPRAs) to examine cis and trans effects between human and mouse embryonic cells across thousands of individual orthologous regulatory elements, including enhancers, promoters of protein-coding genes, and promoters of long non-coding RNAs (lncRNA) genes. Their approach revealed that cis effects are widespread across eRNAs, lncRNAs, and mRNAs, trans effect are less common and generally weaker than cis effects. In addtion, trans effects are specific to eRNAs, suggesting that the redundancy of eRNAs enable them to buffer trans effects. The authors also identify 44 TFs that are associated with trans effects. Importanly, they found that cis-trans compensation is uncommon within individual regulatory elements. The enrichment of cis-trans compensation at conserved lncRNA and mRNA TSSs but at eRNAs or non-conserved TSSs suggests that inter-enhancer compensation, rather than intra-enhancer (cis-trans) compensation, is a widespread feature of mammalian genomes. Overall, I found this study revealed interesting findings to uncover how gene expression and mammalian regulatory elements evolves. However, I do have a couple of major comments.

Major comments
1.        To identify the differentially expressed TFs between hESCs and mESCs, the authors apply edgeR directly to the CPM values of orthologous genes between human and mouse. Although the authors admitted that the differences between hESCs and mESCs may be reflective of differences in isolation and culture conditions in the section of Discussion, using an appropriate control to normalize the gene expression in human and mouse, respectively, is necessary.
2.     The authors apply a bunch of linear regression models followed by a likelihood ratio test to determine the contribution of a single motif to the MPRA activity. However, the underlying mechanisms of using absolute values of cis effect size and not using the absolute effect sizes for the trans model should be elaborated. Why the motif class instead of the motif itself is included in the regression analysis of cis effect. Also, as for the cis effects, would the disruption of certain motifs impede the co-binding of other transcription factors? Would different TF-TF interaction modes also contribute to the differential MPRA activity in human and mouse?

Minor comments
1.      The reason of using CMV promoter as a positive control should be provided.
2.     In some of the statistical significance evaluations (e.g. Fig 3A), one-sided Mann Whitney test, rather than two-sided test, should be used.
3.     In the supplementary motif analysis, the reason of using Box-Cox transformation needs to be explained.
4.     On page 10, line 30: between the human BCAR3-AS1 TSS and region, 'and' should be removed.
5.     Page 12, line 10: absort -> absorb.
6.     Page 18, line 49-50: inter-enhancer compensation between cis and trans effects dominates. 'between cis and trans effects' should be removed.

**Authors’ response to reviewers**

***Below we present a point-by-point response to the reviewers regarding the manuscript***

***“Analysis of regulatory element evolution between human and mouse reveals a lack of***

***cis-trans compensation”, which now has a new title: “Cis and trans effects differentially***

***contribute to the evolution of promoters and enhancers.” The reviewer comments were***

***extremely helpful in clarifying and bolstering some of our results. Their insightful***

***suggestions also helped us to better contextualize our findings. We believe our***

***manuscript has improved significantly. Our replies to the comments are outlined below,***

***in blue text. We have also highlighted any resulting changes to our manuscript and***

***supplement in blue, as well.***

**Reviewer #1:**

This paper uses MPRA to compare activity of fragments overlapping transcription start sites

from human and mouse in both human and mouse embryonic stem cells. From these data,

divergence in both cis- and trans-regulation was inferred. I think this paper is a strong

contribution to the field. It was also the most refreshing paper I've read in quite some time; it is

beautifully and clearly written throughout (with the few exceptions noted below), making what

can be a complicated topic accessible to a broad audience. I want to thank the authors for this

care in preparing their work. A concrete example of this clarity is explaining the numbers of how

many fragments they started and ended up with and how they got from one to the other. This

degree of clarity is rare and greatly appreciated. Figures are also nicely constructed and convey

information clearly.

We thank the reviewer very much for these kind words.

Despite my strong enthusiasm for this work, I take issue with two core conclusions presented,

as described in more detail below. I also offer suggestions for other ways to improve the work.

Primary critiques: 1. I do not think that the conclusion stated prominently in the title, abstract,

figure 6 title, and discussion, that these data argue that compensatory cis and trans effects are

"rare" at individual regulatory elements and in contrast to other data in the field, is wellsupported

by the data. First, overall, 40% of elements affected by both cis and trans divergence

showed compensatory effects. While 40% is not the majority, I would also not describe it as

"rare". Second, and perhaps even more important given the way this result is presented as

being in conflict with previously published work, when only the data for conserved mRNAs are

considered (which is the most comparable to many of the previous RNA-seq based studies that

drew conclusions about compensatory evolution), the percentage of genes with compensatory

effects increases to over 50% (~65%? eyeballing last bar in Figure 6C). I would certainly not call

that rare. And it looks consistent to broad conclusions from prior work to me. Finally, these

conclusions are based on 10s of regulatory elements in this study, but thousands of genes in

the prior work. Indeed, the sentence on page 16, line29/30 says "Conversely, conserved

lncRNA and mRNA TSSs showed mostly compensatory effects". I find this in stark contrast to

the title "… reveals a lack of cis-trans compensation". Especially since I think most people

thinking about gene expression are thinking about expression of mRNAs (and maybe lncRNAs)

much more than eRNAs. Pg 18, lines 29-32 is one example of a conclusion I don't think is

consistent with the mRNA data presented, which is the subset of the current data most similar to

prior work studying effects at the whole-gene level.

We thank the reviewer for bringing this to our attention, and upon reflection, we agree with the

reviewer that some of the language we used was not precise and needed to be clarified.

Specifically, we now clarify that while we do see *cis-trans* compensation at conserved gene

Response to Reviewers Click here to access/download;Personal Cover;rebuttal.docx

promoters—and that this is consistent with previous studies—we do not see *cis-trans*

compensation at enhancers, including those that are conserved. These clarifications, which we

have made throughout the entire manuscript, note our contributions to the field more explicitly.

The changes we have made to address this are highlighted below:

1. We changed the title of our manuscript to more explicitly reflect the data. It is now “*Cis*

*and trans effects differentially contribute to the evolution of promoters and enhancers.*”

2. We changed the Abstract to be explicit about the fact that we see the lack of *cis-trans*

compensation at enhancers, but not conserved promoters: “While we found that this *cistrans*

compensation is common within promoters, we did not see evidence of widespread

*cis-trans* compensation at enhancers.”

3. We altered the relevant results section to explicitly state the same: “Consistent with

previous results, we found that the majority of conserved mRNAs with both *cis* and *trans*

effects showed compensatory *cis-trans* effects (58%, **Figure 6B**). Conserved lncRNAs

also showed an excess of *cis-trans* compensation (57%, **Figure 6B**). However,

conserved enhancers showed an excess of directional *cis-trans* effects (75%, **Figure**

**6B**), as did non-conserved TSSs (aggregated: 72%, **Figure 6B**). Thus, whereas mRNA

and lncRNA TSS conservation is associated with *cis-trans* compensation, regulatory

element turnover between human and mouse is associated with directional *cis-trans*

effects. Moreover, enhancers are associated with directional effects, regardless of

conservation status.”

4. We changed the Discussion to reflect this as well: “When assessing *cis-trans*

contributions at regulatory elements rather than genes, we find that conserved gene

promoters—both lncRNA and mRNA TSSs—indeed show an excess of *cis-trans*

compensation. Non-conserved gene promoters show less *cis-trans* compensation.

Similarly, a recent publication showed that, when looking at TF binding across different

mouse strains, *cis-trans* compensation was only enriched in conserved TF binding

sites28. Interestingly, we do not find excessive *cis-trans* compensation at enhancers. In

fact, both conserved and non-conserved eRNAs show an enrichment of directional *cistrans*

effects (**Figure 6B**).”

5. We also changed the Conclusion: “Notably, we find that while compensation between *cis*

and *trans* effects is common at conserved mRNA and lncRNA TSSs, it is much rarer at

enhancers. Our results support the idea that compensation across enhancers—rather

than within individual enhancers—is a widespread feature of mammalian genomes.”

We believe that these changes have substantially clarified where our results are consistent with

previous work, while explicitly noting our novel contributions to the field.

2. I also take issue with the strength of the data (at least as I understand it) underlying the

claims about redundant regulatory elements. Figure 6G-H take nearby (within 1Mb) additional

regulatory elements (enhancers and TSSs) into account, finding that there is less compensatory

divergence within one of the elements they test when there are other elements nearby. The then

propose that this is because the neighboring elements might help regulate the gene and provide

another mechanism for compensation. I have no problem with presenting this model as

speculation (as is done at some points), but on pg 16, line59 an assumption is made that the

nearby FANTOM5 regulatory elements being considered are redundant with the focal fragment

that I do not see justified. That is, there might be neighboring elements, but most might NOT be

redundant, which undermines the model presented. Pg. 18, line 45 is an example of this: "we

find that highly redundant regulatory elements are less likely to show compensatory…” To me,

“redundant regulatory elements” implies something like shadow enhancers, that have very

similar activity in space, time, and expression level. But the data used for this analysis does not

seem to take the activity of nearby regulatory elements into account, so I don’t think saying

“redundant” is justified. This is also a key feature in the model presented in Figure 7. To be

clear, I think it is fine to present this model, but the assumptions, and the appropriateness of the

data used to test them, should be stated more explicitly.

We thank the reviewer for this constructive commentary. As a result, we have performed a more

robust analysis to test our hypothesis that elements showing directional *cis-trans* effects are

surrounded by more neighboring “redundant” enhancers. Previously, as the reviewer noted, we

had not been explicitly testing for enhancer redundancy, only overall numbers of enhancers.

Now, we have performed a more principled analysis to specifically identify “redundant”

enhancers, and our hypothesis holds: elements showing directional *cis-trans* effects are

surrounded by more neighboring enhancers whose activities are correlated across >1800

FANTOM5 samples.

The analysis we performed is as follows:

1. Instead of looking within an arbitrary window of 1 Mb, we downloaded the topologicallyassociated

domains (TADs) that have been experimentally determined in hESCs and

mESCs by Dixon et al., 2012 (1). TADs define the “operational limit” of enhancerpromoter

contacts and are thus a much more principled boundaries to use.

2. For every element in the MPRA, we calculated the pairwise correlation between the

CAGE activity of the element of interest and the CAGE activity of neighboring enhancers

in the same TAD. These pairwise correlations included 1828 FANTOM5 samples.

3. To find the number of “redundant” enhancers within the same TAD, we used a modified

version of Otsu’s binarization technique (2) to threshold the distributions of pairwise

correlations into those that are “non-redundant” and those that are “redundant”.

An outline of the analysis is shown below, which now comprise new subpanels of **Figure 6**:

*Example of Otsu’s binarization technique, used to subset enhancers within the same TAD (shown in gray in schematic) into those that are nonredundant and redundant. The threshold is denoted by the vertical line and the “redundant” enhancers are those to the right of the threshold, which in this case includes 49 enhancers. This approach is done individually for each element in the MPRA, so it has the advantage of not being subject to biases that may exist across different elements or TADs.*

We reasoned that if the number of “redundant” enhancers is higher for those showing directional

*cis-trans* effects, it should be stronger in conserved elements (i.e., elements with a CAGE peak

in both human and mouse), as inter-species conservation serves as a proxy for biological

relevance. Indeed, conserved elements showing directional *cis-trans* effects are surrounded by

more redundant enhancers within the same TAD. These data are now new subpanels of **Figure**

**6**:

*The total number of enhancers within the same TAD (top) and the number of correlated “redundant”*

*enhancers within the same TAD (bottom), separated by the conservation status of elements in*

*the MPRA. Only elements with both cis and trans effects are plotted. P values shown are from two-sided Mann Whitney tests.*

We also wanted to check if the general trend we see above holds across all biotypes. So we

separated out each biotype element into compensatory and directional, and again asked: how

many nearby correlated enhancers are associated with each class of *cis-trans* effect? While

statistically underpowered after performing this separation, the trend of directional elements

having more “redundant” enhancers in the same TAD also holds across different conserved

biotypes, as we show in new supplemental **Figure S14**:

*Although the p-values from two-sided Mann Whitney tests are higher due to smaller sample sizes, across all biotypes, elements showing directional cis-trans effects have higher numbers of*

*redundant enhancers than elements showing compensatory cistrans effects. Only conserved biotypes are shown.*

Finally, we noticed that our MPRA library included some elements within the same TAD. So as

to ensure our results were not biased by over-representation of particular TADs, we randomly

down-sampled our data to include only 1 element from each TAD 1000 times and calculated

bootstrapped 95% confidence intervals. The down-sampled data is consistent with the

aforementioned results: conserved elements showing directional *cis/trans* effects have more

redundant elements within the same TAD than conserved elements showing *cis/trans*

compensation (now supplemental **Figure S15**):

*Distribution of median differences between the number of redundant enhancers in directional elements compared to compensatory elements for non-conserved elements (A) and conserved elements (B). Distributions were drawn from down-sampling the data such that there is only one element per TAD represented, randomly 1000 times. Numbers greater than 0 (denoted with*

*the dashed black line) mean that there were more median surrounding redundant elements for those with directional effects compared to those with compensatory effects in that sample. In each graph, the 95% confidence interval is shown (dotted lines); in each plot one of the bounds overlaps with 0 and is hidden behind the dashed line.*

We believe these analyses substantially strengthen our original claim that elements with

directional *cis-trans* effects tend to be more redundant. We have made significant changes to

our Results section to include these new analyses, while being explicit about what our definition

of “redundant” means. We have also removed any mentions of “highly redundant” and replaced

them simply with “redundant.” Combined with the changes made to address the previous point

(that enhancers are specific in their enrichment of directional *cis-trans* effects), we believe our

conclusions have been significantly clarified.

Other comments:

1. The definitions used for cis and trans in the abstract and introduction are not correct. For

example, take this sentence from the abstract: "Whereas cis effects are caused by genetic

variants in close proximity to the target gene, trans effects are due to distal genetic variants that

affect diffusible elements such as transcription factors". Although the proximaly/distal

characterization is often used to classify eQTL as likely to be cis or trans acting, it does not

actually define the effects of these variants. cis-effects are those that arise from a genetic

variant affecting expression of a gene located on the same chromosome (cis = same side), and

trans-effects arise from a genetic variant that can affect expression of a gene on a different

(including the homologous) chromosome. The distinction between cis and trans also doesn't

have to do with linkage disequilibrium, as mentioned in the introduction, although it is true that

cis variants are more likely to be in LD because they are more likely to be close to the affected

gene, but a variant being in LD with the affected gene is not sufficient to show that it is cis

acting. It is possible for a variant to be in LD with the affected gene and act in trans as well as

for a variant not to be in LD with the affected gene but to still act in cis. Given that this study

doesn't use proximal/distal characterization or rely on LD to classify effects as cis or trans, I

suggest you simply eliminate these terms from the manuscript to avoid confusion. (And yes, I

realize that other published papers also unfortunately use similarly incorrect and/or imprecise

definitions….)

We thank the reviewer for bringing this to our attention. We have addressed this with the

following changes in the text:

1. The relevant sentence in the abstract now reads: “Whereas *cis* effects are caused by

genetic variants located on the same DNA molecule as the target gene, *trans* effects are

due to genetic variants that affect diffusible elements and thus may impact target genes

located on any chromosome.”

2. Similarly, the relevant sentences in the introduction now read: “*Cis* effects are due to

genetic variants that are on the same chromosome as the target gene; for example,

genetic variants located in gene promoters or enhancers that affect transcription factor

(TF) binding sites. Conversely, *trans* effects are driven by diffusible elements (such as

TFs) and can therefore be on any chromosome relative to the genes they affect.”

These changes have eliminated all mentions of “proximal” or “distal” to classify effects and have

also eliminated any mentions of linkage disequilibrium.

2. Citations: Key papers foundational for this work are not cited. For example, Wilson et al.

(2008) Science, in which mice carrying human chromosomes were used to disentangle cis- and

trans-regulatory effects is very related to this work, yet not cited. In addition, a review paper

(Signor et al) is cited in the introduction for the method rather than the original Wittkopp et al.

(2004) paper where it was first described. In general, I think it is fine to cite a review when a

point is made that is drawn from a large collection of studies, but for statements where the

evidence comes from a small number (1-5?) of original research papers, I think it is more

appropriate to cite the original works. Also, in the opening sentence, although the King and

Wilson 1975 paper is often credited with proposing the importance of transcriptional regulation

for evolutionary divergence, Britten and Davidson lay this argument out first and more clearly in

their 1969 paper.

We absolutely acknowledge that these should be cited and have added all suggested

references and associated context to the Background/Introduction:

1. We were not aware of the Britten and Davidson paper and thank the reviewer for noting

this oversight. We have changed the first sentence of the introduction to move away

from crediting any one specific group, and now reference both papers: “Since it was

suggested over half a century ago that changes in transcriptional regulation underlie

phenotypic differences between species (3,4), it has become clear that changes in gene

expression are heritable and often play a role in the evolution of phenotypes.”

2. We have changed the Signor et al. review citation (5) to the original Wittkopp et al. 2004

paper (6) where F1 hybrids were first described. We have also added additional citations

to more recent original work assessing intra- and inter-specific variation in *cis* and *trans*:

Coolon et al. 2014 (7) and Metzger et al. 2017 (8) as well as an early study that

addresses *cis-trans* compensation: Landry et al. 2005 (9).

3. We added a citation to the Wilson et al. 2008 work (10) which we agree is very related to

our work. After discussing allele-specific hybrid studies in the Background section, we

added the following sentence: “Such hybrid methods have even been used to assess

gene expression divergence between humans and mice (10), although such an

approach in distantly related species is limited to examining a single artificially-inserted

chromosome.”

3. Figure 2C - I would love to see a figure added to the main text that directly compares the

magnitude of the cis effect measured in hESC to that measured in mESC for the same

fragment. For example, a scatterplot, with one axis expression in hESC, the other expression in

mESC, and each point the cis-effect measured for each fragment in the two cell types.

We thank the reviewer for this suggestion. We have added a scatter plot showing the measured

*cis* effects in hESCs and mESCs, which is now **Figure 2D**:

*Scatter plot showing the cis effect sizes in hESCs and mESCs for all 1644 active regulatory elements in the MPRA. Those with significant cis effects in hESCs are orange, in mESCs are green, in both are black, and in neither are gray. Spearman’s rho is shown.*

Similarly, we have also added a scatter plot showing the measured *trans* effects in human and

mouse sequences, which is now **Figure 4D**:

*Scatter plot showing the trans effect sizes in human sequences and mouse sequences for all 1644 active regulatory elements in the MPRA. Those with significant cis effects in human are orange, in mouse are green, in both are black, and in neither are gray. Spearman’s rho is shown.*

These new plots further highlight a result that we have emphasized in the paper: *cis* effects

dominate over *trans* effects.

4. Page 6, Line 8 "mimicked endogenous activity levels" is a bit misleading. I initially thought

you had compared expression driven by each fragment to that driven by the native gene. But,

then realized you were just talking about the endogenous levels at the level of biotype group

(eRNA, lncRNA, mRNA) rather than individual genes. Please rephrase.

We have removed this confusing terminology and simplified the sentence as follows: “As

expected, all TSS biotypes were more active than negative controls, and eRNAs had the lowest

activity across biotypes while mRNAs had the highest activity.”

5. Pg 10, line30 TYPO: "the human BCAR3-AS1 TSS and region and the region shown in

Figure 3)"

We have fixed this typo.

6. Pg 12,line 6 TYPO: "may allow for them to absort trans effects at minimal fitness costs."

We have fixed this typo.

7. Pg. 18, line 18 - please explain more what you mean by "…larger accumulation of trans

effects in eRNAs is likely due to their higher redundancy".

We have clarified the sentence as follows: “Indeed, the larger accumulation of *trans* effects in

eRNAs is likely due to their higher redundancy: previous work by the FANTOM5 consortium has

shown that on average, genes only have ~2 TSSs but are regulated by ~5 enhancers (11).”

8. Pg 19, last paragraph - one additional caveat to this study should be mentioned: that the

activity of regulatory elements was assayed from transfected plasmids rather than elements

integrated into the genome functioning in their native genomic context. This difference likely

affects the activity of the elements compared to the context in which they evolved.

We have added the following sentence to the last paragraph of the Discussion: “Finally, as

MPRAs measure the activities of regulatory elements via transiently transfected plasmids, future

work is needed to probe these elements in their native genomic context in which they evolved.”

9. Reference list: Danko et al is shown as reference 4 and 13.

We have fixed this mistake in the reference list.

10. Pg 3 line 22 - text says you need F1 hybrids for this analysis, but you also need parents to

infer trans-regulatory differences with this strategy.

We have rephrased the sentence to clarify this as follows: “One of the most common

approaches has been to perform allele-specific RNA sequencing of two parental strains and

their corresponding F1 hybrid offspring, which can separate regulatory variants acting in *cis*

(which show allele-specific effects in the hybrid) from those acting in *trans* (which affect both

hybrid alleles equally).”

11. Pg 3, line 27-31 - oversimplifies and misrepresents the literature on cis- and trans-regulation

and compensation; please go back to original papers for this work to look for intricacies that

should be more faithfully represented and cited here (original research, rather than review).

We acknowledge that our overview of prior work was too brief. We have expanded the section

to more faithfully represent prior work, and also included additional citations of original papers:

“In general, these hybrid studies have shown a predominance of *cis* effects between species

(6,10,12–14), with *trans* effects playing a larger role within species (7,8,12,15). Moreover, *cis*

and *trans* effects were found to often occur simultaneously and affect target gene expression in

opposite directions (7–9,16). This so-called “compensation” between *cis* and *trans* effects is

thought to be a result of stabilizing selection on gene expression over evolutionary time

(7,8,16).”

12. Pg 6, line 24 - please include in the main text how the 1644 active sequences are distributed

among eRNAs, lncRNAs, and mRNAs

We added the following clarification to the main text, immediately following the description of the

1644 sequences: “When doing biotype-specific analyses, we focused on the set of 1,262 active

sequence pairs (523 mRNAs, 471 lncRNAs, and 268 eRNAs) that could be reliably assigned to

the three main biotypes (see methods).”

We also added the following clarification to the Supplemental Methods, Biotype Assignment

section which explains why active sequences are left out of the biotype analyses: “If an element

was assigned to different biotypes in human and mouse, we referred to it as a “biotype switch”

and removed it from biotype-specific analyses, as we were not confident in its annotation.”

13. Figure 2 B make X-axis labels consistent in the mESC and hESC plots. Also adjust

transparency of points to make grey points more visible.

We have made the x-axis labels consistent in all paired volcano plots (**Figure 2B** and **Figure**

**4B**) and have adjusted the transparency to enhance the grey points. An example is shown here:

*Volcano plots showing cis effects in hESCs and mESCs; x-axis labels are consistent and grey points are more visible.*

14. Pg 12, line 4 - There should be citations to support the claim that "enhancers are often

redundant". I don't think the literature as a whole supports the "often" part of this sentence. I'd

say "can be" or "are sometimes" redundant is a more accurate statement of our understanding.

See literature on shadow enhancers and redundant enhancers.

We have changed this sentence to use the phrasing “enhancers are sometimes redundant”, and

also added the FANTOM5 enhancer paper citation (11), which shows that genes, on average,

are targeted by ~5 enhancers compared to ~2 TSSs (which we also mention in the Discussion,

as outlined in point 7 above).

15. Pg 14, line 7 & line21 - The language to describe the set sizes of TFs is confusing here. "We

found 137 TFs were significantly associated with trans effects" & "Of the 120 TFs we found to

be associated with trans effects". I assume the second number reflects the final set size after

including the RNAseq data. The section seems to just need a lead in clause to clarify why this

number has changed.

We thank the reviewer for bringing this to our attention. We have added the following clarifying

sentence: “Of the 137 TFs we found to be significantly associated with *trans* effects, 120 were

one-to-one orthologs detected in our RNA-seq data.”

**Reviewer #2:**

In this study, the authors systematically performed Massively Parallel Reporter Assays (MPRAs)

to examine cis and trans effects between human and mouse embryonic cells across thousands

of individual orthologous regulatory elements, including enhancers, promoters of protein-coding

genes, and promoters of long non-coding RNAs (lncRNA) genes. Their approach revealed that

cis effects are widespread across eRNAs, lncRNAs, and mRNAs, trans effect are less common

and generally weaker than cis effects. In addtion, trans effects are specific to eRNAs,

suggesting that the redundancy of eRNAs enable them to buffer trans effects. The authors also

identify 44 TFs that are associated with trans effects. Importanly, they found that cis-trans

compensation is uncommon within individual regulatory elements. The enrichment of cis-trans

compensation at conserved lncRNA and mRNA TSSs but at eRNAs or non-conserved TSSs

suggests that inter-enhancer compensation, rather than

intra-enhancer (cis-trans) compensation, is a widespread feature of mammalian genomes.

Overall, I found this study revealed interesting findings to uncover how gene expression and

mammalian regulatory elements evolves.

We thank the reviewer very much for these kind words.

However, I do have a couple of major comments.

Major comments

1. To identify the differentially expressed TFs between hESCs and mESCs, the authors apply

edgeR directly to the CPM values of orthologous genes between human and mouse. Although

the authors admitted that the differences between hESCs and mESCs may be reflective of

differences in isolation and culture conditions in the section of Discussion, using an appropriate

control to normalize the gene expression in human and mouse, respectively, is necessary.

We thank the reviewer for this constructive comment. We now realize that we had not been

sufficiently clear in explaining our RNA-seq analysis methodology. Notably, we are not directly

comparing CPMs; rather, we are comparing the edgeR trimmed mean of M-values (TMM)

normalized gene counts. This method is consistent with validated published pipelines to

compare inter-species differential expression (17–19). Moreover, to address the reviewer’s

concerns, we have performed additional sanity checks to ensure that our differential expression

analysis is robust to potential statistical artifacts. We therefore believe that our list of

differentially expressed TFs is statistically valid, though our limitations regarding culture

conditions already mentioned in the discussion hold.

Specifically, we have taken the following steps to address this comment:

1. We clarified our Methods, RNA-seq analysis section to more comprehensively

characterize our pipeline and its appropriateness: “We normalized gene expression

values using the trimmed mean of M-values (TMM) normalization method in edgeR,

similar to previous cross-species comparisons (17,18). Briefly, TMM normalization rescales

samples relative to each other under the assumption that most genes are not

differentially expressed (20). This assumption is valid when comparing human and

mouse ESC expression, because even between more distant mammals—such as

humans and opossums—gene expression is tightly correlated (19). To find differentially

expressed genes, we used the edgeR-limma pipeline (20) (filtering out any genes with

normalized cpm < 1) to model paired samples (transfected and untransfected) and

control for transfection status. For plotting purposes, we quantified gene expression in

tpm units in each transfected sample using DESeq2 (21).”

2. We have performed additional sanity checks on our RNA-seq data, which now comprise

a new supplemental **Figure S11**:

a. We determined that our normalized CPMs are extremely similar across samples,

indicating that the TMM normalization worked as expected (**A**, below).

b. One confounder of cross-species RNA-seq analysis using orthologous gene

assignments can be gene lengths: if these change substantially between

species, it can introduce biases in read quantification (22). However, we found

that the gene lengths of TFs that we determine to be significantly differentially

expressed between hESCs and mESCs are highly similar (**B**, below).

c. We confirmed that the differentially expressed TFs have higher foldchanges than

a set of commonly used housekeeping genes whose expression should be more

constant across species (**C**, below).

*A: TMM-normalized CPMs across samples including transfected samples (dark colors) and untransfected controls (lighter colors) in both hESCs and mESCs. B: gene lengths in human*

*and mouse are similar for differentially expressed TFs, which are outlined in red. C:*

*significantly differentially expressed TFs have higher absolute foldchanges between human*

*and mouse ESCs compared to a set of commonly used housekeeping genes: GAPDH,*

*ACTB, ATP5PB, PKG1, and B2M*

2. The authors apply a bunch of linear regression models followed by a likelihood ratio test to

determine the contribution of a single motif to the MPRA activity. However, the underlying

mechanisms of using absolute values of cis effect size and not using the absolute effect sizes

for the trans model should be elaborated. Why the motif class instead of the motif itself is

included in the regression analysis of cis effect.

We thank the reviewer for raising such questions regarding the *cis* effects model. We have

clarified these issues in the text. Briefly, we use the absolute value of the *cis* effect size because

this reduced model outperformed the one using raw *cis* effect sizes (see below). Moreover, we

recognize that using the term “motif class” was confusing—it actually does contain information

about the specific motif and how it changes between human and mouse sequences. We have

therefore ensured that this parameter is always referred to as “motif disrupted” when mentioned.

We have made the following relevant changes to our manuscript:

1. We have added this explanation to our Supplemental Methods, Finding motifs

associated with *cis* effects section: “As *cis* effects are between two sequences, we first

tested two reduced models: one using the absolute value of *cis* effect size and one using

the raw *cis* effect size (where higher values mean the mouse sequence has more

activity). The reduced models included the mean GC and CpG content across each pair

of sequences and the difference in GC and CpG content between each pair of

sequences (mouse minus human). In the absolute value of *cis* effects model, we also

used the absolute value of the GC and CpG differences. The two models were therefore:

(1) | 𝑐𝑖𝑠 𝑒𝑓𝑓𝑒𝑐𝑡 𝑠𝑖𝑧𝑒 | ~ 𝑚𝑒𝑎(𝐺𝐶) + 𝑚𝑒𝑎𝑛(𝐶𝑝𝐺) + |Δ(𝐺𝐶)| + |Δ(𝐶𝑝𝐺)|

(2) 𝑐𝑖𝑠 𝑒𝑓𝑓𝑒𝑐𝑡 𝑠𝑖𝑧𝑒 ~ 𝑚𝑒𝑎(𝐺𝐶) + 𝑚𝑒𝑎𝑛(𝐶𝑝𝐺) + Δ(𝐺𝐶) + Δ(𝐶𝑝𝐺)

The first reduced model outperformed the second (p-value of 4.05e-11 compared to

1.67e-8), so we therefore chose to move forward with the more robust model using the

absolute value of *cis* effect sizes.”

2. We have removed any mentions of the term “motif class” in both the main Methods and

Supplemental Methods sections. The parameter is now referred to as “motif disrupted”

and explained as follows in the supplement: “we broke sequence pairs up into three

categories: those where the motif is not present in either species (“not present”), those

where the motif is maintained in both species (“maintained”), and those where the motif

is only present in one species (“disrupted”).”

Also, as for the cis effects, would the disruption of certain motifs impede the co-binding of other

transcription factors? Would different TF-TF interaction modes also contribute to the differential

MPRA activity in human and mouse?

This is a very interesting point. Indeed, disrupting certain motifs (or multiple motifs at once)

could impede co-binding of other TFs and we would register it as a *cis* effect. However, as

human and mouse regulatory sequences are often quite diverged, we do not have the power we

need to disentangle co-binding from single binding events and can only look for average

enrichment of motifs. While out of the scope of this current project, perhaps an MPRA across

more species, including some that are more closely related, could begin to address this

question.

It is also true that different TF-TF interaction modes that are DNA-binding independent would be

registered as *trans* effects in our assay. It would be very interesting to look at how differential

TF-TF interaction modes between species contribute to *trans* effects, perhaps by combining

methodologies like yeast two hybrid screens with MPRAs. We have noted the TF-TF interaction

mechanism with an additional sentence in our differential expression section: “We speculate

that a subset of the TFs enriched in *trans* effects that are not differentially expressed may be

contributing to *trans* effects through alternative mechanisms, such as evolutionary differences in

TF-TF interactions.”

Minor comments

1. The reason of using CMV promoter as a positive control should be provided.

Absolutely. We have added a parenthetical to clarify our reason for using CMV as a positive

control: “We also included randomly-generated sequences as negative controls (with 3

barcodes each) as well as tiled regions of the cytomegalovirus (CMV) promoter (which is known

to have high activity in MPRAs across diverse cell lines (23)) as positive controls (with 60

barcodes each), resulting in a final library of 181,065 unique oligonucleotides.”

2. In some of the statistical significance evaluations (e.g. Fig 3A), one-sided Mann Whitney test,

rather than two-sided test, should be used.

We agree, and we have replaced the two-sided test with the one-sided test where appropriate.

Specifically, we replaced it where we had an *a priori* expectation of the results: **Figures 1E, 3A,**

**and 6E**. Everywhere else, we kept the two-sided test results, as we did not have any *a priori*

expectations and it is more conservative.

3. In the supplementary motif analysis, the reason of using Box-Cox transformation needs to be

explained.

We agree with the reviewer. To clarify, the Box-Cox transformation is useful for transforming

non-normal variables (such as those that have skewed positive values, like MPRA activity and

absolute *cis* effect sizes) into normal distributions. We have explained the usage of the Box-Cox

transformation in the Supplemental Methods as follows:

1. In the Finding motifs predictive of MPRA activity section, we added the following

sentence: “We then Box-Cox transformed the mean MPRA activity values to “normalize”

the distribution—without this transformation, since all MPRA activity values are positive

and the distribution is skewed, the assumption of a normal distribution, which is required

for accurate linear regression, is validated.”

2. In the subsequent Finding motifs associated with *cis* effects section, we added the

following clarification: “We then Box-Cox transformed the absolute *cis* effect sizes to

“normalize” the distribution, since as in the MPRA activity model, all values are positive

and skewed.”

4. On page 10, line 30: between the human BCAR3-AS1 TSS and region, 'and' should be

removed.

We have fixed this typo.

5. Page 12, line 10: absort -> absorb.

We have fixed this typo.

6. Page 18, line 49-50: inter-enhancer compensation between cis and trans effects dominates.

'between cis and trans effects' should be removed.

We have removed these words.

Bibliography

1. Dixon JR, Selvaraj S, Yue F, Kim A, Li Y, Shen Y, et al. Topological domains in

mammalian genomes identified by analysis of chromatin interactions. Nature. 2012 Apr

11;485(7398):376–80.

2. Otsu N. A Threshold Selection Method from Gray-Level Histograms. IEEE Trans Syst Man

Cybern. 1979 Jan;9(1):62–6.

3. Britten RJ, Davidson EH. Gene regulation for higher cells: a theory. Science. 1969 Jul

25;165(3891):349–57.

4. King MC, Wilson AC. Evolution at two levels in humans and chimpanzees. Science. 1975

Apr 11;188(4184):107–16.

5. Signor SA, Nuzhdin SV. The Evolution of Gene Expression in cis and trans. Trends

Genet. 2018 Apr 18;34(7):532–44.

6. Wittkopp PJ, Haerum BK, Clark AG. Evolutionary changes in cis and trans gene

regulation. Nature. 2004 Jul 1;430(6995):85–8.

7. Coolon JD, McManus CJ, Stevenson KR, Graveley BR, Wittkopp PJ. Tempo and mode of

regulatory evolution in Drosophila. Genome Res. 2014 May;24(5):797–808.

8. Metzger BPH, Wittkopp PJ, Coolon JD. Evolutionary Dynamics of Regulatory Changes

Underlying Gene Expression Divergence among Saccharomyces Species. Genome Biol

Evol. 2017 Apr 1;9(4):843–54.

9. Landry CR, Wittkopp PJ, Taubes CH, Ranz JM, Clark AG, Hartl DL. Compensatory cistrans

evolution and the dysregulation of gene expression in interspecific hybrids of

Drosophila. Genetics. 2005 Dec;171(4):1813–22.

10. Wilson MD, Barbosa-Morais NL, Schmidt D, Conboy CM, Vanes L, Tybulewicz VLJ, et al.

Species-specific transcription in mice carrying human chromosome 21. Science. 2008 Oct

17;322(5900):434–8.

11. Andersson R, Gebhard C, Miguel-Escalada I, Hoof I, Bornholdt J, Boyd M, et al. An atlas

of active enhancers across human cell types and tissues. Nature. 2014 Mar

27;507(7493):455–61.

12. Wittkopp PJ, Haerum BK, Clark AG. Regulatory changes underlying expression

differences within and between Drosophila species. Nat Genet. 2008 Mar;40(3):346–50.

13. Tirosh I, Reikhav S, Levy AA, Barkai N. A yeast hybrid provides insight into the evolution

of gene expression regulation. Science. 2009 May 1;324(5927):659–62.

14. Shi X, Ng DW-K, Zhang C, Comai L, Ye W, Chen ZJ. Cis- and trans-regulatory divergence

between progenitor species determines gene-expression novelty in Arabidopsis

allopolyploids. Nat Commun. 2012 Jul 17;3:950.

15. Emerson JJ, Hsieh L-C, Sung H-M, Wang T-Y, Huang C-J, Lu HH-S, et al. Natural

selection on cis and trans regulation in yeasts. Genome Res. 2010 Jun;20(6):826–36.

16. Goncalves A, Leigh-Brown S, Thybert D, Stefflova K, Turro E, Flicek P, et al. Extensive

compensatory cis-trans regulation in the evolution of mouse gene expression. Genome

Res. 2012 Dec;22(12):2376–84.

17. Sudmant PH, Alexis MS, Burge CB. Meta-analysis of RNA-seq expression data across

species, tissues and studies. Genome Biol. 2015 Dec 22;16:287.

18. Ramsey SA. A Method for Cross-Species Visualization and Analysis of RNA-Sequence

Data. Methods Mol Biol. 2018;1702:291–305.

19. Chen J, Swofford R, Johnson J, Cummings BB, Rogel N, Lindblad-Toh K, et al. A

quantitative framework for characterizing the evolutionary history of mammalian gene

expression. Genome Res. 2019;29(1):53–63.

20. Robinson MD, McCarthy DJ, Smyth GK. edgeR: a Bioconductor package for differential

expression analysis of digital gene expression data. Bioinformatics. 2010 Jan

1;26(1):139–40.

21. Love MI, Huber W, Anders S. Moderated estimation of fold change and dispersion for

RNA-seq data with DESeq2. Genome Biol. 2014;15(12):550.

22. Smid M, Coebergh van den Braak RRJ, van de Werken HJG, van Riet J, van Galen A, de

Weerd V, et al. Gene length corrected trimmed mean of M-values (GeTMM) processing of

RNA-seq data performs similarly in intersample analyses while improving intrasample

comparisons. BMC Bioinformatics. 2018 Jun 22;19(1):236.

23. Mattioli K, Volders P-J, Gerhardinger C, Lee JC, Maass PG, Melé M, et al. Highthroughput

functional analysis of lncRNA core promoters elucidates rules governing tissue

specificity. Genome Res. 2019 Jan 25;29(3):344–55.

**Second round of review**

**Reviewer 1**

Already pasted in the form above, but duplicating here just in case.

The authors have done an excellent job with this revision, addressing my primary concerns about the conclusions drawn from the work as well as the strength of the data supporting the model presented relying on redundant enhancers. The introduction is now much better referenced and more fairly depicts that state of the field. The switch from an arbitrary 1 MB window to one based on TAD structure is also an improvement, as is incorporating CAGE activity estimates for other enhancers predicted within each TAD. I have only a one more major and a few more minor lingering comments:

Major:
I finally put my finger on what was bothering me about the use of enhancers/eRNAs in various places during both rounds of reviewing this manuscript. It is that when I think of enhancers, I think about the effects they have on mRNA expression by working together with a basal promoter rather than RNA transcripts produced from the enhancer sequences themselves (eRNAs). These two fundamentally different elements of enhancers often feel muddied to me in the manuscript. For example, when I read in the abstract "we found that trans effects are rare but stronger in enhancers than promoters" I think this means the effects of the enhancer on mRNA expression, but it doesn't. In the methods, it says: "As eRNAs have two TSSs, we included both of its TSSs and both of its TSSs' orthologous sequences in the pool." I understand from this that the assay was for activity of the TSS controlling production of eRNAs rather than the activity of the enhancer controlling mRNA expression jointly with the gene's promoter. I think the latter is what is more often meant when people write about enhancers and their evolution. To the best of my knowledge, no relationship has been established between the amount of eRNA transcribed from an enhancer and the impact of an enhancer on expression of the associated mRNA. To avoid confusion, I think you should write about eRNAs rather than enhancers in nearly all places where the word 'enhancer' is currently used. Another example of this problematic phrasing is online 359: "by focusing on enhancers, lncRNA promoter, and mRNA promoters…" I think this would be more accurate and less confusing if written as "by focusing on sequences driving expression of eRNAs, lncRNAs, and mRNAs". I think similar changes are needed elsewhere and looking carefully at every use of the word enhancer to determine if it would be more accurately written as eRNA or eRNA promoter is necessary.  

One example of why I think this distinction is important can be found on lines 377-380. The publication cited is talking about the effects of TF binding on the enhancer's regulation of mRNA, not eRNA expression, yet the lower levels of cis-trans compensation reported here for 'enhancers' is really for TSSs driving expression of eRNA. Consequently, I think this is an apples to oranges comparison.  


More minor:
1. Unless I missed it, CAGE is never defined or described.

2. The abstract from the Andersson et al. 2014 FANTOM paper clarifies: "We further explore the utility of enhancer redundancy, which explains gene expression strength rather than expression patterns". I think including a similar clarification in this paper that you are talking about strength (expression level) rather than expression pattern would also be good.

3. line 110 — although the revisions improved the sentences to this section, I think the use of the word "recapitulate" here is still too strong and misleading. If I understand better based on the revision, you confirmed they are expressed, but no that they recapitulate their normal expression. Please rephrase to avoid using this word here.

4. Title to Figure 6. Delete "Only". Let the reader decided if 40% is a lot or a little.

5. Line 37 - delete the word "equally". Trans can impact both alleles but do so unequally because of cis-reg differences.

6. Not critical to address in the paper, but I was confused in the response to reviewers of how an element biotype switching would arise biologically. Do you think this is improper annotation in one species or that there is truly a mechanistic change between species?

7. Line 35: The language is not specific enough here, allele-specific RNAseq does not "separate regulatory variants" but rather separates the proportion of expression variation attributable to cis- and trans-regulatory variation. I know this is what the authors meant but it reads like this approach resolves individual variants which it does not.

**Reviewer 2**

The authors have fully addressed all my concerns. The manuscript has been substantially improved and I have no further comments.

**Authors’ response to reviewers**

We have addressed all the minor edits requested by the editors
